# The Role of Lymphatic Endothelial Cells in Liver Injury and Tumor Development

**DOI:** 10.3389/fimmu.2016.00548

**Published:** 2016-11-29

**Authors:** Veronika Lukacs-Kornek

**Affiliations:** ^1^Department of Medicine II, Saarland University Medical Center, Homburg, Germany

**Keywords:** lymphatic endothelial cells, lymphatics, liver injury, HCC

## Abstract

Lymphatics and lymphatic endothelial cells (LECs) possess multiple immunological functions besides affecting immune cell migration, such as inhibiting T cell proliferation and antigen presentation by dendritic cells. Moreover, they control the trans-endothelial transport of multiple molecules and antigens. Emerging evidence suggest their active involvements in immunregulation, tumor, and metastases formation. In the liver, increased lymphangiogenesis, specifically at the portal area has been associated with multiple liver diseases in particular primary biliary cirrhosis, idiopathic portal hypertension, and liver malignancies. Nevertheless, the exact role and contribution of LECs to liver diseases are poorly understood. The review summarizes the current understanding of LECs in liver diseases.

## Liver as a Lymphoid Organ

The liver primarily operates as a metabolic center to maintain homeostasis that includes processing of gut-derived nutrients, the clearance of toxins, and the production of the bile (1). Besides these well-known functions, it is also considered as a lymphoid organ (2). This is on one hand due to the fact that non-parenchymal cells, such as hepatic stellate cells (HSCs) and liver sinusoidal endothelial cells (LSECs), take on antigen presenting and immunomodulatory functions to create a tolerant microenvironment (2, 3). On the other hand, the liver encompasses large populations of resident immune cells, such as Kuppfer cells, NK, T, and NKT cells that shape the local immune response, respond to danger signals and closely interact with parenchymal and non-parenchymal liver cells (3). These resident immune cells are located within the sinusoids where the mixture of arterial and venous blood carrying oxygen and gut-derived metabolic products arrives into the liver. From the sinusoids blood flows toward the central vein and finally leaves the liver conveying blood to the vena cava inferior. It is less known about the lymphatic circulation of the liver despite of the fact that it produces between 25–50% of the total lymph received by the thoracic duct (4, 5). This review summarizes the current understanding of the lymphatics of the liver and their known functions under steady state and during liver injury. Liver injuries manifest in various diseases including autoimmune hepatitis, infectious [hepatitis C virus (HCV)- and hepatitis B virus (HBV)-induced liver hepatitis], and metabolic disorders. Major causes of metabolic injuries are alcoholic liver damage (manifesting in liver steatosis, hepatitis, and cirrhosis) and the diet-related non-alcoholic fatty liver disease (NAFLD). Biliary injuries involve primary sclerosing cholangitis and primary biliary cirrhosis that are considered as immune-mediated liver disorders. Independent of the diverse etiology, liver inflammation and damage trigger a wound healing process that progressively leads to liver fibrosis, cirrhosis, and end-stage liver disease (6).

## The Lymphatic System of the Liver

The hepatic lymphatic system is divided into a deep and a superficial fraction (5, 7). The former follows the hepatic vein and the portal tracts, and the later collects lymph from the convex and inferior surfaces of the liver. The lymph itself originates in the perisinusoidal space of Disse (4). At the hepatic sinusoids, the interstitial space contains collagen fibers that connect LSECs and hepatocytes and form the portal limiting plate. Thus, fluid from the sinusoids flows through this structure and moves toward the perilobular space (it is referred as the space of Mall) and finally enters the portal lymphatic vessels (4, 5, 7, 8). This fluid movement is attainable due to the hydrostatic pressure differences observed between the portal vein and the interstitial space (9). Additionally, because of pressure gradient between arterial capillaries and the interstitial space, some blood is filtered through the peribiliary capillaries that surround the interlobular bile ducts. Nevertheless, the contribution of this process for the total liver lymph output is less than 10% (10). Besides the above-described route, the interstitial fluid can also follow the interstitial space connected with the hepatic capsule that contains superficial lymphatic vessels (5, 7). Both, the deep and superficial lymphatics of the liver drain primarily to the hepatic/celiac lymph nodes (LNs) (7, 11).

## Lymphatic Content and Cellular Transport

The lymph generated in the perisinusoidal space contains 80% of the proteins present in plasma (5). The content of the lymph gains increasing attention as it contains self-peptides derived from intracellular, membrane-associated, and matrix proteins (12, 13). Moreover, it carries apoptotic cellular materials, infectious agents and represents a remote communication system for small molecules (e.g., cytokines) and cell-derived vesicles between the organ and its draining LN (13–16). The relevance of small molecule/vesicle trafficking *via* the lymphatics to the etiology of liver diseases is entirely unexplored. Such self-antigen delivery can be a key in autoimmune liver diseases. Moreover, biliary content during bile obstruction leaks to the lymphatics at the portal tract (5, 17) and probably reaches the draining LNs. Since bile acids might trigger inflammatory responses and necroptosis, it could influence hepatic immune responses arising within the draining LN.

Due to the resident immune, parenchymal and non-parenchymal cells, a tolerogenic environment is created for immune responses within the liver (3). Nevertheless, if immunity is required as a response to for example pathogens either monocyte-derived DCs present in intrahepatic myeloid-cell aggregates for T cell population expansion (iMATEs) provide bases for efficient T cell responses (18) or cytotoxic T lymphocytes are generated by migratory DCs reaching the draining LN (19, 20). On the other side of the immune spectrum, migratory DCs are likely involved in the generation of regulatory T cells toward dietary antigens in the liver-draining LN (21). The lymphatics thus represent a crucial channel for a potential immunogenic and tolerogenic response outside of the liver suppressive environment (19, 22). To ensure this function, the lymph transports various immune cells. Accordingly, electron microscopy studies revealed the presence of DCs in between the limiting plate of hepatocytes and in the interstitial space of portal tract (4). This migratory process is more active after LPS injection (4). Not only liver resident but also circulating DCs can enter the lymphatic system in the liver, and this DC blood-lymph translocation seems to alter DCs and creates a more tolerogenic phenotype under steady state (23, 24). This could be due to DC interaction en route with liver non-parenchymal cells such as LSECs (25) or with lymphatic endothelial cells (LECs) along the lymphatics (26). Thus, the lymphatic circulation of liver-resident DCs and the circulating DC translocation might contribute to important peripheral tolerogenic responses under steady state. The major migratory cell population is the cDC1 (CD11c^+^CD103^+^CD11b^−^) cells, and it remains to be elucidated whether monocyte-derived DCs or cCD2 (CD11c^+^CD103^−^CD11b^+^) cells contribute to the migratory cell population under differing circumstances (20, 27).

DC migration is maintained by CCR7–CCL21 interaction, where CCL21 is secreted by LECs that are also positive for various adhesion molecules and glycoproteins that are involved in cellular transport, such as gp38, ICAM-1, and E-selectin (28, 29). Besides LECs, EM study revealed the presence of fibroblast-like cells close to collagen fibers at the portal area representing fibroblastic reticular cells (FRCs) (4). Migratory DCs display close correlation with FRCs near the portal tract (4). Accordingly, in human liver, a low number of gp38^+^ FRCs are present at the portal area under steady state (30). FRCs secrete CCL19 that guides DC migration and provide survival factors for immune cell homeostasis (28, 31). Importantly, under pathological conditions, such as in primary biliary cirrhosis, the portal FRC and LEC network extends and is associated with structures similar to tertiary lymphoid organs (30). Similarly, in murine *P. acnes*-induced granulomatous hepatitis, portal tract-associated lymphatic structures, so called PALTs, are formed where T and B cell responses arise (32). Further studies are necessary to clarify that such tertiary lymphoid structure formation is related to migratory and lymphatic changes in liver diseases or represent a pathological structure where LN-independent immune responses influence disease progression.

Besides DCs, lymphocytes, plasma cells, and mast cells could be identified within the lymphatic vessels of the liver and near the portal tract under steady state (4, 5). While memory lymphocytes and plasma cells are common travelers within lymphatic vessels, the exact function of mast cells remains uncertain within the healthy liver. The later is especially intriguing, since mast cells release inflammatory mediators during various liver diseases and contribute as accessory cells to disease progression (33). The liver is especially rich in lymphocytes involving not only conventional T cells but also innate lymphoid cells that express lymphoid homing markers, such as CCR7 (34, 35). Nevertheless, future studies are necessary to determine to which extent the various lymphocyte subpopulations travel *via* the lymphatics from the liver and what are the biological consequences of their migration.

## Lymphatic Endothelial Cells of the Liver

Lymphatic endothelial cells are the building blocks of lymphatic capillaries and vessels and express variety of molecules that distinguish them from blood endothelial cells (BECs) such as CCL21 or cadherin-13 (Table 1) (29, 36). Most of these molecules refer to LECs within secondary lymphoid organs (SLO); however, some differences due to the liver environment could be observed (Table 1). For example, lymphatic vascular endothelial hyaluronan (Lyve-1) is specific for LECs in lymphoid organs but is present in LSECs and in some liver macrophages (37). The best way is to identify liver LECs based on their expression of CD31 and gp38 (podoplanin). Liver LECs are CD45^−^CD31^+^gp38^+^ and thus can be distinguished from FRCs (CD45^−^CD31^−^gp38^+^), from LSECs (CD45^−^CD31^+^gp38^−^), and from the recently described gp38^+^ liver progenitor cells (CD45^−^CD31^−^CD133^±^gp38^+^) (38).

**Table 1:** **Summary of surface markers for identifying murine and human lymphatic endothelial cells**.

Endothelial markers (LECs and BECs)	Endothelial markers excluded from BECs
ICAM-1 (CD54)	Lyve-1^a^
CD44	Prox-1
	VEGFR3
CD31	CCL21
CD34	Desmoplakin
	Integrin α9, α1
E-, P-selectin	B-chemokine receptor D6
Plakophilin	Cadherin-13
	MMR
	Gp38 (podoplanin)

*^a^Present in liver LSECs and some liver macrophages*.

*BECs, blood endothelial cells; MMR, macrophage mannose receptor*.

Lymphatic endothelial cells not only provide the structural unit for the vessels but also are involved in additional biological processes. As discussed already, *via* its expression of cytokines and adhesion molecules, LECs guide immune cell migration. Additionally, they are active participants in the nearby arising immune responses. They directly diminish DC maturation and T cell proliferation and thus function as a negative regulatory circuit during immune responses (26, 29, 39). A variety of immunoregulatory factors are expressed by LECs that enable these functions. For example, LECs secrete TGFβ and nitric oxide, all of which are immunosuppressive (39, 40). Additionally, LECs lack the expression of co-stimulatory molecules and instead are rich in co-inhibitory markers, such as PDL1 (29, 39, 41, 42).

Lymphatic endothelial cells also possess the ability to express self-antigens and induce CD8 T cell deletion and serve as antigen reservoir for CD4 T cell tolerance (41–43). They also possess surface receptors for endocytotic activity and able to sample from their environment (44). Importantly, most of these immunomodulatory potentials are connected with LECs present in SLO, thus raising the question what are the similarities and differences between SLO-associated LECs and LECs present along the lymphatic vessels. Unfortunately, such comparison studies have not been conducted. It is also uncertain whether liver LECs are able to acquire soluble antigens from the lymph and have antigen-presenting capacity.

Lymphatic endothelial cells are also actively involved in cholesterol homeostasis, and the removal of cholesterol by lymphatic vessels is dependent on the uptake of HDL by scavenger receptor class B type I expressed in LECs (45–47). In line with this, endothelial O-glycan deficiency led to disorganized lymphatic vessels and resulted in the development of fatty liver disease (NAFLD) due to the missing lymphatic removal of gut-derived lipid products (48). Since lipid metabolic changes are associated with various liver diseases, it will be interesting to evaluate in more details how this affects lymphatic function and *vice versa* how lymphatic changes are reflected in liver metabolic alterations.

## Lymphatics and Liver Diseases

### Chronic Liver Diseases

Multiple studies have demonstrated that the lymphatic system is significantly altered during liver diseases. The number of lymphatic vessels as well as the lymphatic flow increases in fibrotic and cirrhotic livers (37, 49–52). This is in line with observations that VEGF-C and VEGF-D expression is elevated during fibrosis (51, 53, 54). More importantly, the increased lymphangiogenesis is positively correlated with disease severity (49, 52). Moreover, the higher flow observed within the lymphatics during liver diseases could have additional consequences. Increased interstitial flow elevates the expression of cell recruiting cytokines (e.g., CCL21) and thus influences immune cell migration toward the draining LN (29). The flow at the same time likely reduces the portal pressure *via* channeling the excess fluid in cirrhosis and in portal hypertension (55).

Increased number of LECs is present during idiopathic portal hypertension (56), HCV-associated cirrhosis (52), and primary biliary cirrhosis (50). Given the wide-range of biological processes where LECs are involved, it is likely that the increase in the number of lymphatic vessels possesses functions exceeding fluid handling. The inflammatory environment triggers cytokine production in LECs and therefore increases immune cell recruitment (29). Additionally, bacterial products such as LPS (that is increased in portal vein during cirrhosis) induce not only chemo-attracting cytokine production but also can activate Nf-Kb in LECs and thus consequently upregulate Prox1 and VEGFR-3 (57). Both molecules raise the sensitivity to VEGF-C and VEGF-D and thus influence lymphangiogenesis (57, 58). Within the liver, this remains to be elucidated.

### Liver Tumor and Metastases Development

One of the consequences of liver diseases is the development of hepatocellular carcinoma (HCC). Human HCC samples displayed Lyve-1^+^ cells in the tumor-surrounding environment (37), and lymphatic vessels are present in the vicinity of metastatic liver tumors (37, 59, 60). In line with this observation, VEGF-C- or VEGF-D-expressing liver tumors are more prone to spread within the liver (60). The liver metastases of colorectal cancer also exhibit gp38^+^ peri- and intra-tumoral lymphatic vessels that were correlated with tumor growth and metastases potential (61). Accordingly, intrahepatic invasion was the main prognostic marker for colorectal cancer and for intrahepatic cholangiocarcinoma and likely represents the main route of cancer dissemination in the liver (62–64). Indeed, intrahepatic cholangiocarcinoma is often associated with LN metastasis that translates to poorer outcome and reduced patient survival (63).

Lymphatic endothelial cells could facilitate such tumor cell spreading *via* CCL21–CCR7 interaction. Some colorectal cancer cells express CCR7 and thus could migrate toward the homeostatic chemokine CCL21 expressed by LECs (29). Additionally, lymphatic flow-induced chemokine gradient (e.g., CCl21 or CXCL12) could be sufficient to drive metastases of tumors positive for cytokines as observed in gliomas (29, 65). The exact mechanisms for HCC and other liver cancers are not well understood. Similarly, LECs display multiple immunomodulatory roles within the tumor microenvironment. LECs induce the recruitment of regulatory T cells, alter features of tumor-associated stroma, and contribute to the immunosuppressive milieu favoring tumor growth (29, 66, 67). Additional studies are necessary to evaluate these possibilities also for liver cancers and metastases.

## Summary and Outlook

**Figure 1:**
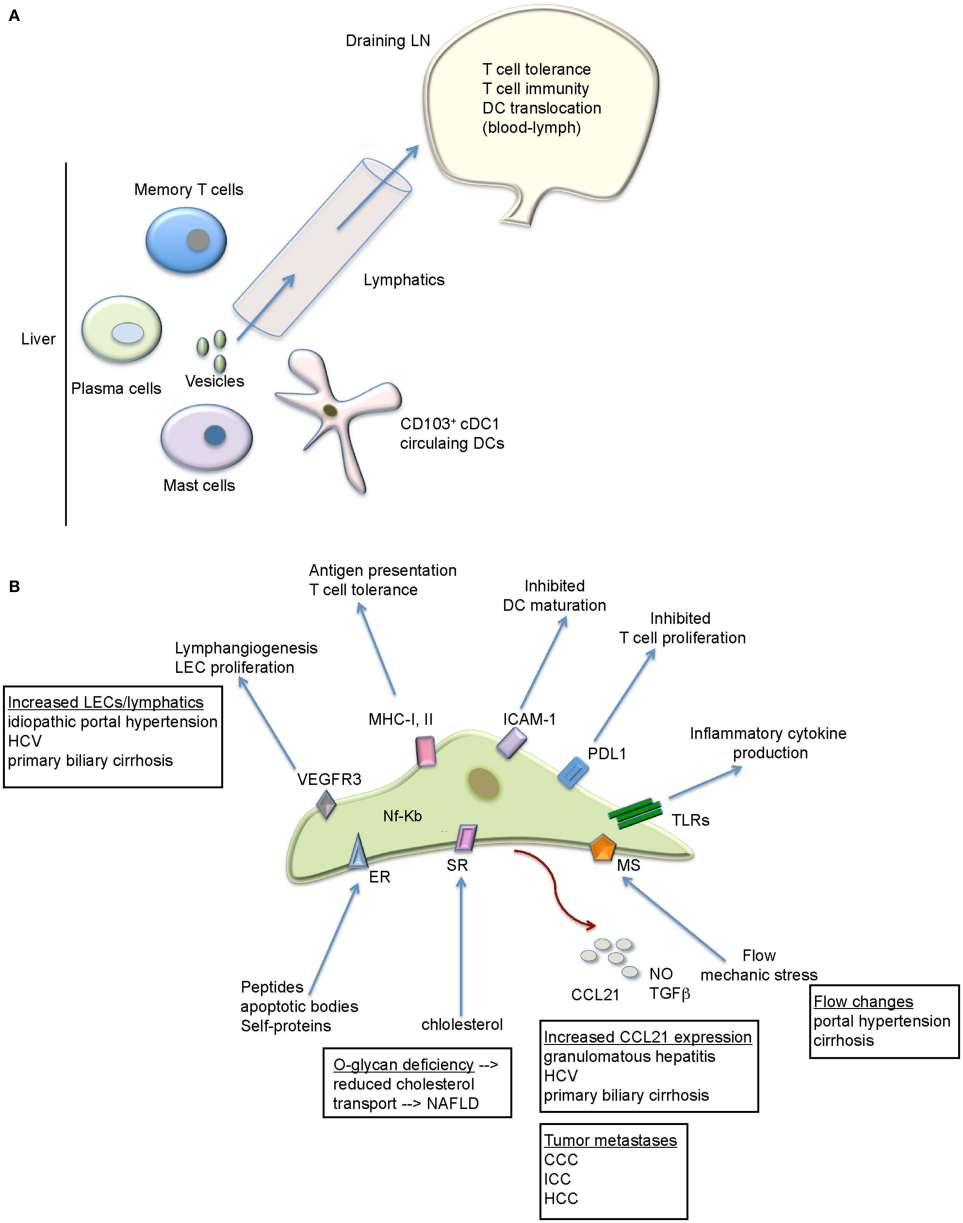
**The functional role of the lymphatics within the liver**. **(A)** Besides fluid handling, the hepatic lymphatics transport various immune cell types, proteins, and vesicles to the draining LN. The LN provides the environment where not only tolerogenic but also immunogenic T cell response can arise that target liver pathogens/antigens. **(B)** LECs exhibit a variety of immunoregulatory functions that might be relevant for liver diseases. Future studies are necessary to evaluate all of these possibilities. Liver diseases with lymphatic/LEC alterations are depicted. CCC, colorectal carcinoma; ER, endocytic receptor; HCC, hepatocellular carcinoma; ICC, intrahepatic cholangiocytes carcinoma; MS, mechanosensors; SR, scavenger receptors, TLRs, toll-like receptors.

Taken together, the liver is a unique metabolic and immunological niche within the body. Its lymphatic system represents a complex anatomical organization with a large lymph output. Based on the repertoire of the biological functions associated with lymphatics and LECs (Figure 1), it is suggested that LEC expansion is not only a passive accompanying event during liver diseases. This is particularly interesting since LEC changes seem to be reflective of the type of peripheral inflammation (68). Thus, this line of research urges more attention and studies that clarify its exact contribution to liver disease pathogenesis. This is possible, as improved marker combinations allow the flow cytometry detection and sorting of these cells from the liver. This, together with other techniques (e.g., histological analyses), provides solid basis for further functional investigations. This could raise our understanding of liver diseases and open novel therapeutic opportunities.

## Author Contributions

VL-K has designed and written the manuscript and prepared the table and the figure.

## Conflict of Interest Statement

The author declares that the research was conducted in the absence of any commercial or financial relationships that could be construed as a potential conflict of interest.

## Abbreviations

CCL21, CC-chemokine ligand 21; CCl_4_, carbon tetrachloride; CCR7, C–C chemokine receptor type 7; CD, cluster of differentiation; cDC1/2, conventional dendritic cells type 1/2; FACS, fluorescence-activated cell sorting; FRCs, fibroblastic reticular cells; HSCs, hepatic stellate cells; LECs, lymphatic endothelial cells; LNs, lymph nodes; MS, mechanosensors; SLO, secondary lymphoid organs; SR, scavenger receptors; TLRs, toll-like receptors.
